# Utilization of in Vitro Anther Culture in Spelt Wheat Breeding

**DOI:** 10.3390/plants8100436

**Published:** 2019-10-22

**Authors:** Csaba Lantos, Szandra Purgel, Katalin Ács, Bernadett Langó, Lajos Bóna, Krisztina Boda, Ferenc Békés, János Pauk

**Affiliations:** 1 Cereal Research Non-profit Ltd., P.O. Box 391, H-6701 Szeged, Hungary; csaba.lantos@gabonakutato.hu (C.L.); szandra.purgel@gabonakutato.hu (S.P.); katalin.acs@gabonakutato.hu (K.Á.); bernadett.lango@gabonakutato.hu (B.L.); lajos.bona@gabonakutato.hu (L.B.); 2 George Oláh PhD School, Budapest University of Technology and Economics, 1521 Budapest, Hungary; 3 Department of Medical Physics and Informatics, University of Szeged, Korányi fasor 9, Szeged 6720, Hungary; boda.krisztina@med.u-szeged.hu; 4 FBFD PTY LTD., Hull Road 34, Beecroft, NSW 2119, Australia; firinc47@gmail.com

**Keywords:** in vitro androgenesis, anther culture, breeding, diallel analyses, haploid induction, spelt wheat

## Abstract

The efficiency of in vitro anther culture was screened in a full diallel population of four spelt wheat genotypes and ten F_1_ hybrids. Genotype dependency was observed based on the data of embryo-like structures (ELS), green-, albino plantlets. In the diallel population and ten F_1_ hybrids, the green plantlets production ranged from 13.75 to 85.00 and from 6.30 to 51.00, respectively. The anther culture-derived plants of F_1_ hybrids were grown up in the nursery. At the harvest, 436 spontaneous doubled haploid (DH) plants were identified among the 1535 anther culture-derived transplanted and grown up individual plants. The mean of spontaneous rediploidization was 28.4% which ranged from 9.76% to 54.24%. In two consecutive years, the agronomic values of ‘Tonkoly.pop1’ advanced line were compared with seven DH lines of ‘Tonkoly.pop1’ in the nursery. The DH lines achieved competitive values in comparison with ‘Tonkoly.pop1’ advanced line based on the 11 measured parameters (heading date, plant height, yield, hardness, width and length of seed, TKW, hulling yield, flour yield, protein and wet gluten content). These observations presage the efficient utilization of anther culture in spelt wheat breeding.

## 1. Introduction

In the last two decades, due to some special advantageous agronomic traits, i.e., wide adaptability, tillering ability, abiotic stress tolerance, and high biomass, the growing area of spelt wheat increased year by year [1,2,3,4]. In the organic farming system, spelt wheat is an attractive species because of the above-mentioned traits. Spelt is a competitive plant species against weeds due to their high tillering ability. Spelt has good adaptation to unfavourable soil conditions with low inputs. Furthermore, the interest in spelt wheat is increasing in human consumption because of the numerous benefits such as high content of protein, minerals (Zn, Cu, Fe), and other bioactive compounds such as dietary fibres, phenolic compounds, phytosterols, and vitamins [3,5,6,7,8,9,10].

Spelt wheat is also grown in the marginal regions of wheat growing area. Implicitly, the capacity of spelt breeding was mitigated in comparison with bread wheat programs in the last period. So, there are many challenges (improvement of lodging resistance, fragile spike, earliness, and yield improvement) in the growing and breeding of spelt wheat to increase the economy of cultivation and produce marketable products [2,4,11].

Recently, many modern approaches exist for breeders which can reduce the long process of breeding and increase the efficiency of breeding programs. The doubled haploid (DH) plant production belongs to these modern biotechnology methods of plant breeding [12,13,14,15,16,17]. The main advantage of DH plant production methods is the development of completely homozygous lines in one generation, while in conventional breeding programs the development of homozygous lines requires more generations. Recessive alleles can be identified and fixed using DH methods in early steps of the breeding process. The DH plant production methods can be combined with other breeding strategies such as mutation or transgenic technologies [18,19].

Homogeneity is one of the essential requirements in the breeding of varieties and hybrids. Homozygous lines can be produced within one generation using biotechnological methods. Well-established DH plant production methods are applicable for breeding and research of some crop plants, for example: Barley, wheat, triticale, maize, and rice [12,13,14,15]. In spite of these benefits, only a few published data can be found in connection with DH plant production of spelt wheat [20,21,22,23,24].

In crop plants, there are three frequently applied DH plant production methods, namely chromosome elimination, anther culture, and isolated microspore culture. In haploid induction of spelt wheat, the chromosome elimination was reported by Escarnot et al. in 2014 [21]. However, because of low efficiency (16.1 embryos/100 pollinated florets and 38 plantlets/100 embryos) this method has not been applied for practical breeding, yet. The isolated microspore culture method is an effective and time-saving method, for example in barley and rapeseed [13,25,26,27]. This method was also tested with four spelt wheat genotypes but the albinism hindered the efficiency of green plant regeneration, the method required further improvements [23], yet. Anther culture was mentioned at first by Schmid in 1990 [20], the average of anther culture-derived plantlets was 0.9 plants/100 anthers in that experiment. Takács et al. [28] also reported the induction of in vitro androgenesis in anther culture of a single genotype (the number of responsive anthers was 6.4%). Unfortunately, these promising results were inadequate for practical breeding programs. 

Recently, the anther culture has proven to be an efficient method via four spelt genotypes (‘Franckenkorn’, ‘GK Fehér’, ‘Mv Martongold’, ‘Oberkulmer Rotkorn’), the green plantlets production ranged from 20.93 to 83.08 green plantlets/100 anthers depending on genotype [23]. Some Spanish and Central European spelt genotypes were also tested in anther culture; the green plant production (1.8–15.6 green plants/100 anthers) was influenced by genotype [24]. However, a high number of albinos was regenerated from the anther culture-derived embryo-like structures (ELS) of these genotypes, the phenomenon of albinism limited the efficiency of anther culture [24].

In this study, our purposes were to screen the responsivity of a range of spelt genotypes in anther culture and verify the efficient application of DH plant production in the breeding procedure of this species. A diallel population of four spelt wheat genotypes and ten breeding targeted F_1_ hybrids was involved in the experiments. The data of the produced ELS, green-, and albino plantlets was collected and analysed. The percentage of spontaneous rediploidization was calculated based on the seed production of anther culture-derived plants of ten F_1_ hybrids. The agronomic values (heading date, plant height, yield, hardness, width and length of seed, TKW, hulling yield, milling yield, protein- and wet gluten content) of selected seven DH lines were monitored based on data of field experiment in two consecutive years. 

## 2. Results

### 2.1. In Vitro Androgenesis in Anther Culture of Spelt Wheat Full Diallel Population

The in vitro androgenesis was induced in the anther culture of each of the tested genotypes, the genotype influenced the efficiency of the method. The number of ELS ranged from 32.75 to 173.33 ELS/100 anthers depending on genotype (Table 1).

The green plantlets production ranged from 13.75 to 85.00 green plantlets/100 anthers depending on genotype (Table 2). The highest value was achieved with the ‘Franckenkorn’/’Martongold’ hybrid. In anther culture of two hybrids (‘Franckenkorn’/’Martongold’ and ‘Oberkulmer Rotkorn’/’Franckenkorn’), these values were significantly higher in comparison with their lower responding parents, respectively.

The phenomenon of albinism was mitigated in anther culture of spelt wheat genotypes (Table 3). The number of produced albino plantlets ranged from 0 to 15 albinos/100 anthers depending on genotype. These values of two hybrids (‘Franckenkorn’/’Martongold’ and ’Franckenkorn’/’GK Fehér’) were significantly lower in comparison with albino plantlets production of ‘Franckenkorn’.

Based on statistical analyses, the genotype influenced significantly the number of produced ELS, green-, and albino plantlets (Table 4). The effects of general combining ability (GCA) were also significant for each tested parameter. Significant reciprocal effect was measured in the number of regenerated green plantlets (Table 5). GCA effects were significant for each parameter of ‘Martongold’, ‘Franckenkorn’, and green plantlets of ’Oberkulmer Rotkorn’ (Table 6).

Some specific combining ability (SCA) effects differed significantly from zero for green (‘Martongold’/’Franckenkorn’) and albino (’Franckenkorn’/’GK Fehér’) plantlets (Table 7). 

After the rooting of the in vitro green plantlets, the green ones were acclimatized in the greenhouse. In October, the acclimatized plants were transplanted and grown up in the nursery. The seeds of spontaneous DH plants were harvested only and propagated in further tests and analysed of the spelt breeding program.

### 2.2. Efficiency of Anther Culture in Ten Breeding Orientated F_1_ Genotypes of Spelt Wheat

**Figure 1:**
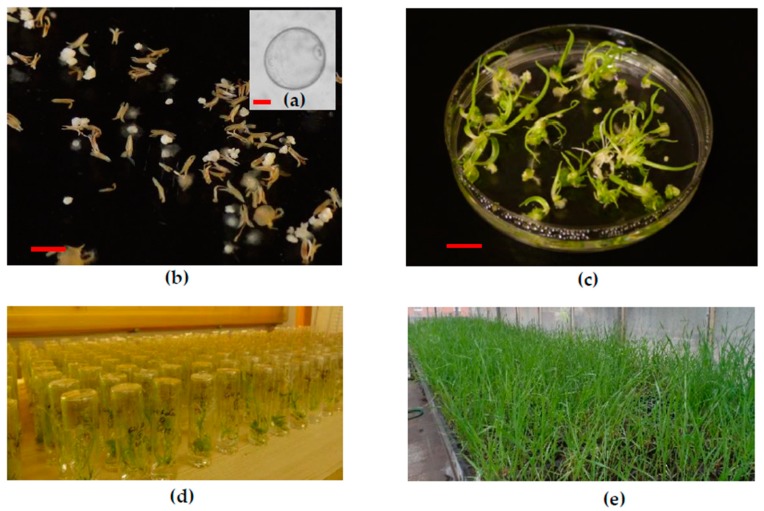
(**a**) Uninucleate microspores at the induction of androgenesis (bar = 10 µm). (**b**) Microspore-derived ELS developed in anther culture after four weeks of cultivation (bar = 5 mm). (**c**) The ELS produced green- and albino plantlets on the regeneration medium (bar = 10 mm). (**d**) The green plantlets were rooted in individual glass tubes, (**e**) which acclimatized to the greenhouse conditions.

The efficiency of anther culture was also tested in the anther culture of ten F_1_ breeding materials. Androgenesis was induced in each of the tested genotypes. The isolated anthers contained uninucleate microspores at the induction of in vitro androgenesis (Figure 1a). The ELS were visible to the naked eye on the fourth week of anther culture (Figure 1b). Genotype influenced significantly the number of ELS, in vitro green and albino plantlets (Table 8). The means of ELS production ranged from 19.5 to 183.47 ELS/100 anthers.

The ELS produced dominantly green plantlets within two weeks on the regeneration medium (Figure 1c). The green plantlets production was influenced by genotype (6.3–51.00 green plantlets/100 anthers). The mean of the in vitro regenerated green plantlets was 28.28 green plantlets/100 anthers. The phenomenon of albinism was observed, on average 4.2–24.3 albinos/100 anthers were regenerated from the ELS depending on genotype. In this experiment, the mean of the albino plantlets production was 11.43 albinos/100 anthers (Table 8).

The in vitro green plantlets were rooted in individual glass tubes (Figure 1d), and the well-rooted plantlets acclimatized to the greenhouse conditions (Figure 1e). The anther culture-derived plants were transplanted in the DH garden of the nursery in October. Altogether, 1535 transplanted plants were grown up until harvest in the nursery. In the DH garden, 436 fertile spelt wheats were identified among the transplanted plants based on the seed production (Table 9). The values of spontaneous rediploidization ranged from 9.76% to 54.24% depending on genotype. The mean of the spontaneous rediploidizaton was 28.40%.

### 2.3. Evaluation of in Vitro Generated Spelt Wheat DH Lines 

‘Tonkoly.pop1’ spelt wheat advanced line as a control and its seven different DH sister lines were compared in the nursery (Table 10). The DH lines showed uniform performance in the field (data not shown). The DH lines achieved similar values than the control in the heading date, plant height, and yield values. The effect of year caused bigger differences on the data of the three measured traits than the effect of genotype. 

Hulling yield, flour yield, and some selected chemical parameters were also evaluated (Table 11). The effect of year was significant on flour yield, protein content, and wet gluten content in both years in several genotypes. The genotype influenced significantly the measured data of hulling yield, protein content, and wet gluten content in both years. In flour yield, significant differences were observed among the genotypes only in the first year.

Some physical parameters of the seeds were also compared in the two-year experiment (Table 12). Kernel hardness of DH lines (9–18) showed significant differences only in the second year, ‘Tonkoly.pop1’ advanced line, and its DH lines could be characterized with soft kernel type. Kernel width values varied in the narrow range, 2.27–2.55 mm, also kernel length was between 7.01–7.44 mm, whilst TKW was between 28.8 and 34.7 g in the selected genotypes. 

## 3. Discussion

Although the methods of plant biotechnology play a key role in modern plant breeding, a few published data are available in connection with in vitro androgenesis of spelt wheat especially from the viewpoint of practical breeding. A full diallel crossing program was carried out with the four varieties (‘Franckenkorn’, ’GK Fehér’, ‘Mv. Martongold’, and ‘Oberkulmer Rotkorn’). Furthermore, ten breeding targeted F_1_ genotypes were involved in our research and breeding program. In F_1_ generation of twenty-two spelt wheat combinations, in vitro anther culture method was tested to produce genetically pure lines for spelt breeding program [22,23,29].

In monocots, some relevant publications reported the genotype dependency and albinism as a bottlenecks of in vitro androgenesis which prohibited the practical application of anther culture in the breeding of more cereal species [12,15,24,30,31,32,33,34]. At the early period of in vitro androgenesis research, the published protocols were not effective for practical spelt breeding programs [20,28]. Recently, anther culture methods have been published as an efficient tool for DH plant production in some spelt genotypes [23,24]. However, the phenomenon of albinism was also mentioned as a limiting factor of anther culture in some experiments [23].

The genotype influenced significantly the efficiency of anther culture in common wheat [35,36,37], and the genotype effect was also published in anther culture of spelt wheat genotypes [20,23,24]. Both reciprocal and nuclear genetic effects were observed in anther culture of common wheat [37]. However, GCA effects were more determinative than the SCA effects for the anther culture parameters [37]. According to relevant publications, the inheritance of anther culture response is determined dominantly by additive genetic effects in common wheat [37,38]. In the present in vitro experiments, a full diallel crossing program and ten F_1_ hybrids were tested in vitro anther culture to clarify the efficiency of this method with a range of spelt genotypes. In vitro androgenesis was induced in each of the tested genotypes, although the genotype influenced significantly the efficiency of the method. In the full diallel population, high number of in vitro green plantlets was produced while the number of albinos was limited. The highest green plantlets production (65.00 green plantlets/100 anthers) was achieved by ‘Franckenkorn’ genotype among the parents similarly to our previous results [23], while ‘Franckenkorn/Martongold’ hybrid produced 85.00 in vitro green plantlets/100 anthers. Based on the statistical analyses, most of the genotypic variance was due to GCA effects; consequently additive genetic variance was a primary contributor to the observed data. Similar phenomenon was reported in common wheat [37,38]. This observation prohibits the practical application of the anther culture in spelt wheat breeding and research.

The ten breeding targeted hybrids produced different quantity green plants depending on genotype. In the nursery, 436 fertile DH lines were identified among the 1535 transplanted plants. The percentage of spontaneous rediploidization was 28.4% which was also influenced by genotype (9.76–54.24%). In our previous experiment, the percentage of spontaneous DH plants was 24.27% (11.8–44.44%) based on data of four genotypes [23]. These values (15–80%) were also influenced by genotype in an anther culture of Spanish and Central European spelt wheat genotypes [24]. In contrast with some critical reviews [12,15,24,30,31,32,33,34], the anther culture was an efficient method for the tested spelt genotypes.

‘Tonkoly.pop1’ advanced line and its seven different DH sister lines were compared in the two-year field experiment. First of all, the spelt wheat DH lines showed uniform performance in the field which is a critical viewpoint in breeding. The DH lines showed competitive values based on their 11 measured parameters (heading date, plant height, yield, hardness, width and length of seed, TKW, hulling yield, flour yield, protein content, wet gluten content) in comparison with the control. In the case of several traits of DH lines, the genotype influenced significantly the measured parameters in both years. Genetically independent DH lines were separated from the advanced line. So, these data emphasized the practical application of DH method in spelt breeding. 

## 4. Materials and Methods

### 4.1. Plant Materials

Four winter type spelt varieties were selected for a full diallel analysis, these genotypes are popular and cultivated varieties in Hungary. ‘GK Fehér’ was released in 2017 by us at the Cereal Research Non-Profit Ltd., while the seeds of other varieties—‘Franckenkorn’, ‘Mv Martongold’, and ‘Oberkulmer Rotkorn’—were supplied to us by the Agricultural Institute, Centre for Agricultural Research, Hungarian Academy of Sciences, Martonvásár, Hungary. A full diallel population (four parents and their 12 hybrids) was generated by crossing of the four spelt varieties. The parents and their F_1_ hybrids were involved in the in vitro experiments. Furthermore, ten breeding targeted F_1_ combinations were also tested in vitro anther culture (Table 13). The parents of the F_1_ combinations were cultivated varieties (‘Franckenkorn’, ‘GK Fehér’, ‘Lajta’, ‘Martongold’, ‘Oberkulmer Rotkorn’) in Hungary, advanced lines (‘Aus’, ‘Bartucz’) or germplasms from Hungarian national gene bank (Center for Plant Diversity’s, Tápiószele: RCAT056296, RCAT058694, RCAT060960).

The donor plants of each genotype were grown in the greenhouse at Cereal Research Non-Profit Ltd., Szeged, Hungary. The vernalized plants were transplanted into 2 l plastic pots which contained a 1:1 peat and sandy soil mix. Volldünger chemical fertilizer (N:P:K:Mg = 14:7:21:1, plus 1% microelements: B, Cu, Fe, Mn, and Zn; produced by Magyar Kwizda Ltd., Budapest, Hungary) were used for fertilizing of the plants once in a fortnight. In the growing period, 20/15 °C day/night temperature were adjusted for plants, respectively. Natural light was supplemented with 3 h artificial light until the collection of donor tillers. For proper plant growth, required fertilizers and fungicides were applied and weeds were removed manually.

### 4.2. Preparation of Anther Cultures

The donor tillers were collected when the microspores were in early- and mid-uninucleate stages. The developmental stages of microspores were checked by Olympus CK-2 inverted microscope (Olympus Ltd., Southend-on-Sea, UK). The collected donor tillers were placed into Erlenmeyer flasks containing tap water and covered by PVC bags to keep high humidity. The collected donor tillers were cold pre-treated at 3–4 °C for 14 days. After the cold pre-treatment, the spikes of the selected genotypes were sterilized in 300 ml 2% NaOCl solution with one drop Tween-80 for 20 min on a shaker. The spikes were rinsed three times with distilled water (Millipore Elix 5) in laminar air flow.

From the prepared spikes, 300 anthers were isolated in each 90 mm diameter glass Petri dishes containing 12 ml ‘W14mf’ induction medium [39,40]. On the first three days of culture, the Petri dish were incubated at 32 °C to apply heat shock treatment. The anther cultures were kept at 28 °C for eight weeks in a dark thermostat.

### 4.3. Plant Regeneration and Acclimatization of Anther Culture-Derived Green Plants

The well-developed ELS with a size of 1–2 mm was transferred into 90 mm diameter plastic Petri dishes (Sarstedt, Newton, MA, USA) which contained the 190–2Cu medium [29]. The ELS regenerated green and albino plantlets on the regeneration medium. The albino plantlets were discarded while the green plantlets with two-three leaves were transferred into individual glass tubes which contained the ‘190–3Cu’ regeneration medium for rooting [23]. 

The well-rooted green plantlets were transferred into the greenhouse. The plantlets were transplanted into plastic plates (66 plantlets/plate) which contained the above mentioned 1:1 soil mix. The plantlets were covered by transparent plastic cover during the 3–4 day-long acclimatization period. The acclimatized plants were grown in the greenhouse following the above-mentioned growing protocol for donor plants in the greenhouse. 

### 4.4. Growing of Anther Culture-Derived Plants in DH Nursery

The acclimatized anther culture-derived plants of the ten F_1_ hybrids were transplanted manually into the DH nursery in October 2018. The transplanted plantlets were irrigated as needed to support the development of roots and acclimatization of the plantlets to the field conditions. The seeds of the spontaneous DH plants were harvested from the fertile plants in July 2019.

### 4.5. Field Study and Evaluation of Spelt Wheat DH Lines

In preliminary experiments, DH lines of ‘Tonkoly.pop1’ advanced line were produced by anther culture. These DH lines were integrated into our breeding program. After DH_1_ generation, seven different individual DH lines of ‘Tonkoly.pop1’ were selected based on their phenotypic and agronomic data (data not shown). These selected seven DH lines along with the control (‘Tonkoly.pop1’ advanced line) were shown in our spelt wheat nursery (40 × 1m plots each) in two consecutive years (2017/2018 and 2018/2019). These DH lines and the control were characterized by some agronomic and grain quality parameters (heading data, plant height, yield, hardness, width and length of seeds, thousand kernel weight (TKW), hulling yield%, flour yield%, wet gluten content) based on the collected data.

Kernel hardness, width, and TKW parameters were measured with a PERTEN SKCS 3100 (Perten Instruments, Sweden) according to the Approved Method [41]. Kernel length was measured with calipers. Protein and wet gluten content were detected by NIR (Mininfra SmarT, Infracont, Hungary). Samples were dehulled with Spelt Huller (Kapacitív Kkt., Hungary), hulling yield was calculated. Dehulled grains were conditioned to 14% moisture content overnight and milled by Brabender Quadromat Senior Mill (Brabender GmbH & Co., Germany) to pass through a 250 µm screen, flour yield was calculated.

### 4.6. Statistical Analyses

Our in vitro experiments were repeated at least three times, minimum 3 × 300 anthers were tested in each treatment. The three important parameters of in vitro androgenesis (number of ELS, in vitro green-, and albino plantlets) were collected and analysed by one-way ANOVA. The data of diallel population were used to determine combining abilities (GCA and SCA) and clarify the reciprocal and nuclear genetic effects in anther culture of spelt genotypes. GCA and SCA and reciprocal effects were analysed using Griffing’s (1956) method 1, model 1 [42]. The percentage of spontaneous rediploidization was calculated based on seed production of anther culture-derived plants of ten F_1_ hybrids. In the field experiment, the measured data of seven DH lines and ‘Tonkoly.pop1’ advanced line were analysed by two-way ANOVA without repetition.

The statistical analyses were carried out using Microsoft Excel 2013 statistical software developed by Microsoft Ltd. (Redmond, WA, USA) and the statistical program SPSS (SPSS Hungary 1115 Budapest Bartók Béla Street 105–113). 

## 5. Conclusions

The spelt wheat breeding program was initialized with a full diallel crossing of four genotypes and ten F_1_ hybrids. In vitro anther culture method was applied in F_1_ generation to support the breeding process. The genotype influenced significantly the androgenic parameters (number of ELS, green-, and albino plantlets). GCA effects were more determinative than the SCA effects in anther culture of the diallel population, anther culture response was determined dominantly by additive genetic effects. In the field experiment, seven anther culture-derived DH lines were tested in comparison with the control genotype. The DH lines were competitive with the control advanced line based on the 11 tested parameters. The anther culture method proved to be an efficient tool to produce lots of DH lines for breeding and applied research in spelt wheat.

## Figures and Tables

**Table 1:** Mean of embryo-like structures (ELS) production anther culture of a full diallel spelt wheat population. Values with the same capital letters are not significantly different (*p* > 0.05).

Genotype		Male Parent			
		‘Oberkulmer Rotkorn’	‘Martongold’	‘Franckenkorn’	‘GK Fehér’
**Female Parent**	‘Oberkulmer Rotkorn’	51.00 b	50.50 b	163.75 ab	120.33 ab
	‘Martongold’	35.25 b	34.25 b	76.00 b	82.25 ab
	‘Franckenkorn’	97.00 ab	155.00 ab	149.50 ab	82.75 ab
	‘GK Fehér’	74.25 b	32.75 b	173.33 a	88.33 ab

**Table 2:** Mean of green plantlets in anther culture of a full diallel spelt wheat population. Values with the same capital letters are not significantly different (*p* > 0.05).

Genotype		Male Parent			
		‘Oberkulmer Rotkorn’	‘Martongold’	‘Franckenkorn’	‘GK Fehér’
**Female Parent**	‘Oberkulmer Rotkorn’	20.75 c	24.00 c	56.25 ab	44.67 bc
	‘Martongold’	19.50 c	19.00 c	35.00 bc	38.50 bc
	‘Franckenkorn’	40.50 bc	85.00 a	65.00 ab	36.25 bc
	‘GK Fehér’	29.25 bc	13.75 c	54.67 b	33.00 bc

**Table 3:** Mean of albinos in anther culture of a full diallel spelt wheat population. Values with the same capital letters are not significantly different (*p* > 0.05).

Genotype		Male Parent			
		‘Oberkulmer Rotkorn’	‘Martongold’	‘Franckenkorn’	‘GK Fehér’
**Female Parent**	‘Oberkulmer Rotkorn’	1.25 b	1.50 b	10.75 ab	8.00 ab
	‘Martongold’	2.75 b	0.00 b	6.00 b	4.25 b
	‘Franckenkorn’	9.25 ab	5.00 b	15.00 a	5.25 b
	‘GK Fehér’	4.50 b	1.50 b	4.00 b	9.00 ab

**Table 4:** Analyses of variance for number of ELS, green-, and albino plantlets.

Source	DF	Mean Squares		
		ELS	Green Plantlets	Albinos
Replications	3	2656.625	99.79	11.875
Genotype	15	9088.233 ***	1465.384 ***	64.967 ***
Error	45	2,117.103	236.415	18.353
Total	63			

*** Significant at *p* < 0.001.

**Table 5:** Analyses of partitioned genotypic variance for ELS, green-, and albino plantlets.

Source	DF	Mean Squares		
		ELS	Green Plantlets	Albinos
GCA	3	6699.7 **	963.34 **	51.786 **
SCA	6	356.5	104.53	12.529
Reciprocals	6	1973.8	330.28*	2.182
Error	45	529.2	59.10	4.588

*, ** Significant at *p* < 0.05 and 0.01, respectively.

**Table 6:** General combining ability (GCA) effects for ELS, green-, and albino plantlets in a full diallel spelt population.

Varieties	ELS	Green Plantlets	Albinos
‘Oberkulmer Rotkorn’	−11.25	−6.4844 **	−0.5937
‘Martongold’	−29.0937 **	−6.7344 **	−2.8750 ***
‘Franckenkorn’	39.2188 ***	16.2656 ***	3.2813 ***
‘GK Fehér’	1.125	−3.0469	0.1875

**, *** t-test is significant *p* < 0.01, *p* < 0.001, respectively; this effect is significantly different from zero.

**Table 7:** Specific combining ability (SCA) effects for (**a**) embryoid, (**b**) green-, and (**c**) albino plantlets.

**a, Genotype**	**‘Martongold’**	**‘Franckenkorn’**	**‘GK Fehér’**
‘Oberkulmer Rotkorn’	−8.40625	10.78125	15.75
‘Martongold’		13.75	−6.15625
‘Franckenkorn’			−3.96875
**b, Genotype**	**‘Martongold’**	**‘Franckenkorn’**	**‘GK Fehér’**
‘Oberkulmer Rotkorn’	−3.48438	0.150875	8.057625
‘Martongold’		12.01563**	−2.53663
‘Franckenkorn’			−6.19238
**c, Genotype**	**‘Martongold’**	**‘Franckenkorn’**	**‘GK Fehér’**
‘Oberkulmer Rotkorn’	0.09375	1.8125	1.15625
‘Martongold’		−0.40625	0.0625
‘Franckenkorn’			−4.34375 **

** Significant at *p* < 0.01.

**Table 8:** Efficiency of anther culture in ten breeding-orientated F_1_ spelt genotypes. Values with the same capital letters are not significantly different (*p* > 0.05).

Genotype	Number of ELS/100 Anthers	Number of Albinos/100 Anthers	Number of Green Plantlets/100 Anthers
Spc24	110.60 ab	4.60 b	39.80 ab
Spc28	163.60 ab	4.20 b	51.00 a
Spc31	183.47 a	17.60 ab	37.20 ab
Spc34	98.80 b	8.10 b	35.40 b
Spc36	66.20 bc	11.70 b	14.30 c
Spc39	109.20 b	24.30 a	17.30 c
Spc40	55.30 bc	9.10 b	6.30 c
Spc42	169.20 ab	22.27 a	25.73 bc
Spc43	19.50 c	4.20 b	8.40 c
Spc50	126.10 ab	8.20 b	47.40 ab
Mean	110.20	11.43	28.28
LSD5%=	73.29	8.17	14.51

**Table 9:** Percentage of spontaneous rediploidization in spelt genotypes.

Genotype	Number of Transplanted Plantlets	Number of Fertile Plants (DH)	Percentage of Spontaneous Rediploidization
Spc24	216	42	19.44%
Spc28	306	68	22.22%
Spc31	144	33	22.91%
Spc34	168	67	39.88%
Spc36	41	4	9.76%
Spc39	153	83	54.24%
Spc40	17	5	29.41%
Spc42	126	52	41.27%
Spc43	52	23	44.23%
Spc50	312	59	18.91%
Mean	1535	436	28.40%

**Table 10:** Heading date, plant height, and yield parameters of ‘Tonkoly.pop1’ advanced line (Control) and its doubled haploid (DH) lines based on field experiment in two consecutive years. Values with the same capital letters are not significantly different (*p* > 0.05).

Genotype	Heading Date		Plant Height (cm)		Yield (t/ha)	
	2017/18	2018/19	2017/18	2018/19	2017/18	2018/19
DH1	141 ab	147 c	115 ab	120 a	5.06 ab	5.95 b
DH2	141 ab	147 c	115 ab	120 a	4.74 ab	5.86 b
DH3	142 ab	147 c	115 ab	125 b	4.85 ab	5.85 b
DH4	143 b	147 c	110 a	125 b	4.19 a	5.38 ab
DH5	142 ab	147 c	115 ab	125 b	4.43 a	6.09 b
DH6	143 b	147 c	110 a	115 ab	4.34 a	6.18 b
DH7	141 ab	147 c	110 a	125 b	4.24 a	6.79 b
Control	140 a	147 c	115 ab	130 b	4.37 a	6.13 b
LSD5%	2.5		10.94		1.31	

**Table 11:** Processing efficiency and some selected chemical parameters of ‘Tonkoly.pop1’ spelt wheat advanced line (Control) and its doubled haploid (DH) lines based on field experiment in two consecutive years. Values with the same capital letters are not significantly different (*p* > 0.05).

Genotype	Hulling Yield %		Flour Yield %		Protein Content %		Wet Gluten Cont. %	
	2017/18	2018/19	2017/18	2018/19	2017/18	2018/19	2017/18	2018/19
DH1	67.6 d	66.9 cd	56.53 ab	60.94 bc	13.6 ab	16.0 bc	33.1 ab	35.1 bc
DH2	66.0 bc	66.0 bc	54.00 a	61.45 bc	13.8 ab	16.1 bc	32.8 ab	35.2 bc
DH3	67.6 d	67.2 cd	55.23 ab	60.96 bc	13.8 ab	16.1 bc	32.2 ab	35.1 bc
DH4	66.3 c	66.2 bc	54.59 a	62.18 c	14.0 ab	15.3 bc	32.3 ab	32.9 ab
DH5	67.9 d	67.2 cd	58.28 b	61.69 c	13.6 ab	15.1 b	31.4 a	31.8 ab
DH6	65.7 bc	66.0 bc	57.05 ab	62.68 c	13.0 a	15.1 b	30.5 a	31.7 ab
DH7	63.4 a	64.1 ab	54.84 a	59.71 bc	13.3 a	15.8 bc	30.3 a	34.4 b
Control	65.6 bc	65.0 b	56.5 ab	62.68 c	16.2 bc	16.8 c	35.1 bc	37.4 c
LSD5%	1.2		3.4		1.59		2.91	

**Table 12:** Values of grain physical parameters of ‘Tonkoly.pop1’ spelt wheat advanced line (Control) and its doubled haploid (DH) lines based on field experiments. Values with the same capital letters are not significantly different (*p* > 0.05).

Genotype	Hardness (−)		Width (mm)		Length (mm)		TKW (g)	
	2017/18	2018/19	2017/18	2018/19	2017/18	2018/19	2017/18	2018/19
DH1	16.0 ab	16.0 ab	2.350 a	2.48 a	7.14 b	7.42 d	31.3 a	32.8 a
DH2	13.0 ab	9.0 a	2.390 a	2.49 a	7.19 b	7.38 cd	31.0 a	33.9 a
DH3	16.0 ab	11.0 ab	2.420 a	2.48 a	7.2 b	7.44 d	32.3 a	33.2 a
DH4	19.0 b	14.0 ab	2.270 a	2.47 a	7.15 b	7.4 cd	29.8 a	33.4 a
DH5	17.0 b	13.0 ab	2.370 a	2.55 a	7.2 b	7.35 c	31.0 a	34.6 a
DH6	18.0 b	14.0 ab	2.290 a	2.48 a	7.11 ab	7.39 cd	28.8 a	33.4 a
DH7	14.0 ab	18.0 b	2.340 a	2.53 a	7.1 ab	7.31 c	30.2 a	34.7 a
Control	18.00 b	17.0 b	2.495 a	2.34 a	7.01 a	7.21 bc	32.4 a	29.5 a
LSD5%	7.47		0.28		0.104		5.9	

**Table 13:** The pedigree of F_1_ combinations tested in vitro anther culture.

Code Number	Genotype
Spc24	‘RCAT056296’//’GK Fehér’/’Franckenkorn’
Spc28	‘RCAT058694’/’GK Fehér’
Spc31	‘Aus’/’RCAT058694’
Spc34	‘Aus’/’Lajta’
Spc36	‘Aus’/’GK Fehér’
Spc39	‘Aus’/ ‘RCAT056296’
Spc40	‘Aus’/’Martongold’
Spc42	‘Aus’/’Bartucz’
Spc43	‘Aus’/’Oberkulmer Rotkorn’
Spc50	‘RCAT060960’//’GK Fehér’/’Franckenkorn’
